# Non-Pigmented Ciliary Epithelium-Derived Extracellular Vesicles Loaded with SMAD7 siRNA Attenuate Wnt Signaling in Trabecular Meshwork Cells In Vitro

**DOI:** 10.3390/ph14090858

**Published:** 2021-08-27

**Authors:** Saray Tabak, Valeria Feinshtein, Sofia Schreiber-Avissar, Elie Beit-Yannai

**Affiliations:** Clinical Biochemistry and Pharmacology Department, Ben-Gurion University of the Negev, Beer-Sheva 84105, Israel; sarayt@post.bgu.ac.il (S.T.); shteiman@bgu.ac.il (V.F.); sofia@bgu.ac.il (S.S.-A.)

**Keywords:** extracellular vesicles, exosomes, primary open-angle glaucoma, trabecular meshwork, non-pigmented ciliary epithelium, aqueous humor, SMAD7, Wnt-TGFb2

## Abstract

Primary open-angle glaucoma is established by the disruption of trabecular meshwork (TM) function. The disruption leads to increased resistance to the aqueous humor (AH), generated by the non-pigmented ciliary epithelium (NPCE). Extracellular vesicles (EVs) participate in the communication between the NPCE and the TM tissue in the ocular drainage system. The potential use of NPCE-derived EVs to deliver siRNA to TM cells has scarcely been explored. NPCE-derived EVs were isolated and loaded with anti-fibrotic (SMAD7) siRNA. EV’s structural integrity and siRNA loading efficiency were estimated via electron microscopy and fluorescence. Engineered EVs were added to pre-cultured TM cells and qRT-PCR was used to verify the transfer of selected siRNA to the cells. Western blot analysis was used to evaluate the qualitative effects on Wnt-TGFβ2 proteins’ expression. EVs loaded with exogenous siRNA achieved a 53% mRNA knockdown of SMAD7 in TM cells, resulting in a significant elevation in the levels of β-Catenin, pGSK3β, N-Cadherin, K-Cadherin, and TGFβ2 proteins in TM cells. NPCE-derived EVs can be used for efficient siRNA molecule delivery into TM cells, which may prove to be beneficial as a therapeutic target to lower intraocular pressure (IOP).

## 1. Introduction

Primary open-angle glaucoma (POAG), the predominant form of glaucoma [1,2], is a chronic, degenerative optic neuropathy characterized by progressive visual field loss [3] that leads to irreversible blindness [2]. Race, older age, family history of the disease, myopia, low diastolic perfusion pressure, and elevated intraocular pressure (IOP) are risk factors for POAG, with IOP being the predominant one [4]. IOP is determined mainly by the delicate balance between the production and drainage of the aqueous humor (AH) through the conventional outflow pathway [5]. From the anterior chamber of the eye the non-pigmented ciliary epithelium-NPCE serves as the site of AH production, while the trabecular meshwork (TM), and Schlemm’s canal are the principal locations of AH outflow. Contraction of the ciliary muscle causes expansion of the TM and opening of Schlemm’s canal, which subsequently increases the conductivity of AH through the TM [6]. The canonical Wnt signaling pathway in TM cells could provide insights leading to new glaucoma therapies [7]. The canonical Wnt signaling pathway is a critical regulator of IOP, and plays a role in extracellular matrix (ECM) expression in TM cells through regulation of matrix metalloproteinases’ (MMPs) activity [8,9,10]. The Wnt signaling pathway also involves activating Wnt target genes which inactivates GSK3β by phosphorylation and stabilizes free β-Catenin [11]. β-Catenin is an important component of cadherin-mediated cell adhesion, linking Cadherins to the Actin cytoskeleton via the adaptor protein α-Catenin [12,13]. Elevation of cytosolic β-Catenin levels in TM cells increases the expression of N-Cadherin [14] and K-Cadherin [15] which stabilize cell-cell junction formation [16], making cadherin-mediated adhesions mechanosensitive transmitters of intercellular forces [16].

In-vivo studies on fibronectin matrix remodeling showed that cadherin-mediated cell-cell contact modifies tissue tension and integrin-mediated cell-matrix interactions [17]. TM cells are mechanosensitive structures that play a major role in ocular outflow resistance, therefore, alterations in cadherin-mediated adhesion between cells may affect IOP regulation. Glaucomatous TM tissues have decreased TM cellularity as compared to non-glaucomatous TM. Increased ECM in trabecular beams and in juxtacanalicular tissue results in increased TM stiffness. Increased actin contractility (due to elevated β-Catenin and Cadherins levels), results in increased cross-linked actin networks, and in dysregulation of TM cell signaling pathways, including the Wnt/β-catenin signaling pathway and the transforming growth factor-beta (TGFβ) [18,19].

The cytokine TGFβ2 is an immunosuppressive factor found in elevated levels in glaucomatous patients [17] and may have an essential role in POAG [18,19]. It has been demonstrated in conjunctival fibroblasts that anti-VEGF agents can regulate the TGF-β2 expression. In this regards it is worthy of note that anti-VEGF drugs, used in clinical practice, such as Ranibizumab, Bevacizumab, and Aflibercept are different in terms of molecular interactions when they bind VEGF with and impact also on TGF-β2 modulation [20]. Therefore, characterization of such features can improve the design of novel biological drugs potentially useful in clinical practice. TGFβ2 is known to induce changes in the actin cytoskeleton, alter cadherin-mediated cell-cell interactions, modify ECM remodeling of human TM cells [21,22], inhibit cell proliferation [23], and induce senescence-like changes [24]. Recent studies have found that TGFβ2, which regulates ECM metabolism including fibronectin, collagen, and elastin, was elevated in the AH and TM of glaucoma patients [25,26]. Since TGFβ2 enhances the adhesion of cells through Cadherins and β-Catenin expression in human TM cells [14], we further investigated the crosstalk of TGFβ2 and Wnt signaling in TM cells. The TGFβ2/SMAD pathway is important in the regulation of ECM deposition in the TM [27]. In contrast to receptor-associated SMAD2 and SMAD3, which mediate TGFβ signaling [28], SMAD7 is a critical inhibitor of TGFβ2 signaling in the TM [29], inducing degradation of the TGFβ2 receptor [30,31]. SMAD7 also acts in the nucleus to disrupt the TGFβ-induced functional SMAD-DNA complex formation [32] and prevents TGFβ2-induced gene expression in the TM [29]. SMAD7 plays a significant role in regulating ECM protein expression in the aqueous outflow pathway [26]. Presently, there are better-tolerated drugs to lower IOP and more effective surgical procedures [3,33]. Nevertheless, no treatment avoids irreversible visual impairment, highlighting the importance of early detection. Coca-Prados and Escribano have previously suggested that signal transduction, which takes place between NPCE and TM tissues, is mediated by AH [34]. In addition, extracellular nano-vesicles called exosomes are a major constituent of the AH [35]. Furthermore, Dismuke et al. reported that AH exosomes deliver esRNAs (exosomal RNAs) from the ciliary body to the TM [36]. In earlier studies in our lab, involving human NPCE-derived exosomes’ characterization [37,38], we were able to provide evidence for their role as signaling mediators of the Wnt signaling pathway in TM cells. These EVs demonstrated a specific uptake mechanism [39] influenced by particle interactions [40,41]. We suggested that these native biological nanoparticles mediate the communication between NPCE and TM cells by ECM remodeling [42], supporting a physiological role in IOP regulation. Extracellular vesicles (EVs) and exosomes particularly are natural transport nano-vesicles, cup-shaped, 40–150nm in length and are secreted by most cell types [43]. Exosomes are derived from the luminal membrane of multi-vesicular bodies (MVBs). These vesicles are released to the extracellular medium [44] following MVBs’ fusion to the cell membrane, and carry single-stranded DNA, coding and non-coding RNA, proteins, lipids, and antigen-presenting molecules of the producing cell [45]. Various reports have addressed the concept of using EVs for endogenous gene delivery [46,47]. However, when using EVs for therapeutic applications, it is crucial to deliver exogenous cargoes, such as small interfering RNA (siRNAs), to their specific target tissues or cells. Artificially synthesized, siRNAs are 19–23 nucleotide long double-stranded RNA molecules [48], used for the transient silencing of genes of interest [49]. Effective pharmacological use of siRNA requires carriers that can deliver the siRNA to its intended site of action. Despite the high therapeutic potential of siRNA, its application in clinical settings is still limited due to the lack of efficient delivery methods [50,51,52,53,54]. EVs are receiving more attention concerning drug delivery since several works demonstrated that EVs could deliver siRNA in vitro [55,56] and in-vivo [57]. Various methods for EVs loading with siRNA have been proposed [58], including electroporation of the EVs and transfection of the donor cell with the cargo of interest. Neither method is perfect however [59,60]. Transfection of the donor cell is a time-consuming process involving numerous steps requiring quality assurance [61], while electroporation was shown to adversely affect the EVs’ structural integrity [62]. In the present study, we used electroporation, creating small pores in the EVs membrane, allowing the siRNA to diffuse into the vesicles [63]. Different conditions were tested to reach maximal siRNA loading efficiency. siRNAs are negatively charged—they cannot penetrate hydrophobic cellular membranes and depend on a carrier. We investigated whether NPCE-derived EVs could act as a carrier and deliver genetic material to TM cells. In addition, we tested whether the siRNA delivered via EVs to TM cells down-regulate the expression of the anti-fibrotic target gene, SMAD7. Finally, we studied the effect of SMAD7 silencing on the expression levels of Wnt-TGFβ2 associated genes and proteins. 

## 2. Results

### 2.1. Establishing the Effect of the Electroporation Conditions on NPCE-Derived EVs Size, Concentration, and Membrane Integrity

Electroporation applies an electrical current to the phospholipid bilayer of the exosomes, leading to the formation of temporary pores. Although electroporation leads to beneficial loading of siRNA over chemical transfection, it might lead to exosome instability, reducing the loading capacity. To ensure that the tested parameters for achieving maximal siRNA loading into the EVs did not lead to the structural instability of the NPCE-derived vesicles, we used TRPS technology and Cryo-TEM analysis. 

#### 2.1.1. TRPS Analysis of NPCE-Derived EVs Size and Concentration before and after Electroporation

The size and concentration of EVs following four different electroporation protocols as well as untreated EVs as control were measured using Tunable Resistive Pulse Sensing (TRPS) technology. Our analysis indicated that the most suitable conditions for electroporation of EVs are 400 v, 125 µF, and one electric pulse (Table 1, condition 1). The average size of 73.33 ± 26.51 nm in diameter and 2.25 × 10^12^/mL concentration of NPCE EVs following the detailed conditions for electroporation of condition 1 were closest to the values of the non-electroporated EVs’ control group (with a size of 76.67 ± 11.06 nm and concentration of 1.38 × 10^12^/mL). For the other three groups (Conditions 2–4), a trend of decreases in EVs’ size and concentration were observed compared to the control group. Values are the mean of three measurements for each condition. Characterization of NPCE EVs was previously done using exosomes markers: CD81, Alix, and TSG101, as was determined by and Exo-Check analysis and Western blot analysis [37,39].

#### 2.1.2. Cryo-TEM Analysis of NPCE-Derived EVs Membrane Integrity Following Electroporation

**Figure 1:**
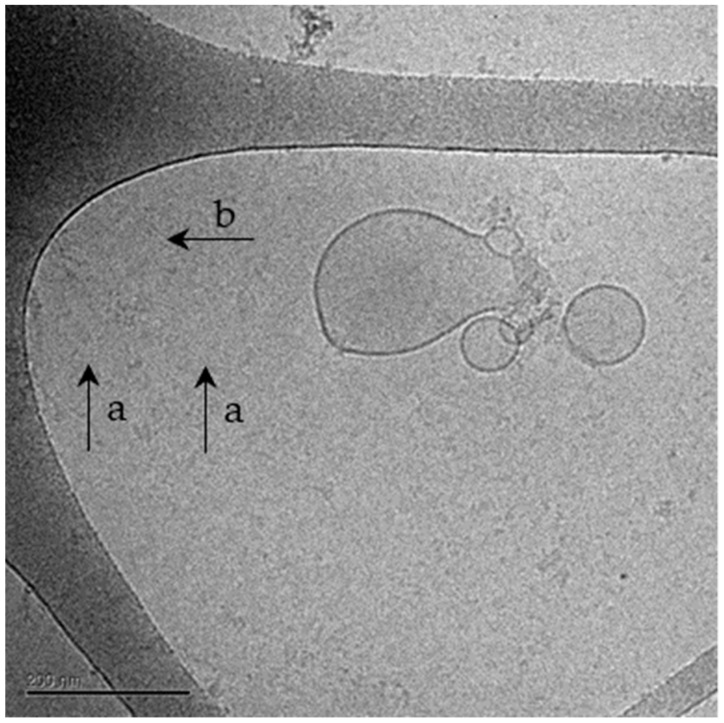
A representative image taken by Cryo-TEM of NPCE-derived EVs following electroporation. Cryo-TEM analysis of NPCE EVs after electroporation. Cryo-TEM analysis was performed to ensure EV’s membrane integrity. An image of the electroporated sample represents the morphological difference between normal, intact EVs (**a**), compared to damaged EVs (**b**).

Cryo-TEM analysis (Table 2 and Figure 1) revealed that the most suitable conditions for electroporation are: 400 v, 125 µF, 0.5 µg/µLEVs, and one pulse in a 0.4 cm cuvette, with 76.02% normal shaped NPCE EVs, maintaining the EVs membrane intact (Table 2, Condition 1) as compared to control.

### 2.2. siRNA EVs Loading Assessment by Fluorescence Analysis

**Figure 2:**
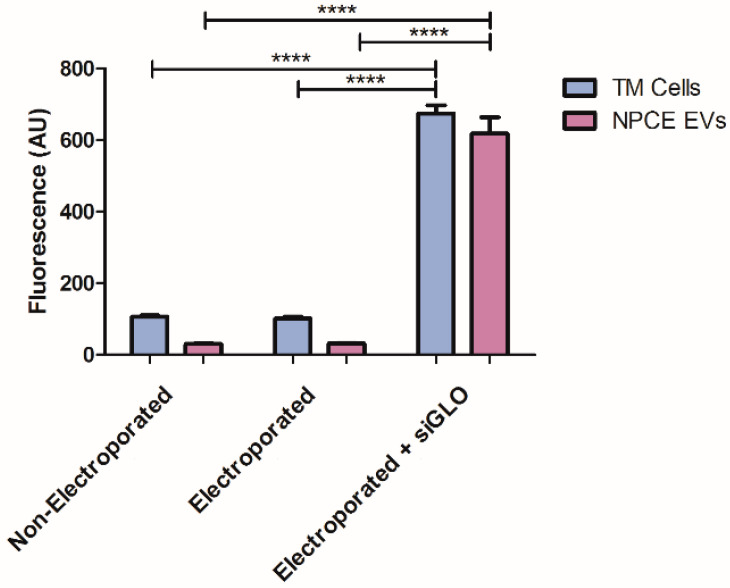
siRNA EVs loading assessment by fluorescence analysis. Testing the encapsulation of fluorescent siRNA in TM cells and NPCE EVs following electroporation. The fluorescent of non-electroporated, electroporated (with no exogenous cargo), and electroporated + siGLO of both TM cells and EVs were evaluated after electroporation. A minimum of three repetition for each group was tested. Two-way ANOVA was used to determine statistical difference, as indicated by asterisks (**** *p* < 0.0001).

A fluorescent oligonucleotide duplex, siGLO green indicator, was used to evaluate siRNA loading into EVs, and EVs uptake by their target TM cells. The fluorescence value of the electroporated sample was 20.26 times higher than the control’s fluorescence, indicating the presence of siRNAs in NPCE EVs. In addition, siRNA was detected in TM cells, with a fluorescence value of that was 6.34 times higher than non-electroporated TM cells. Electroporated TM cells with no siRNA that used as the negative control to eliminate auto-fluorescence of the cells following the electric pulse (Figure 2). 

### 2.3. Detection of siRNA in NPCE-Derived EVs Using Confocal Microscopy

**Figure 3:**
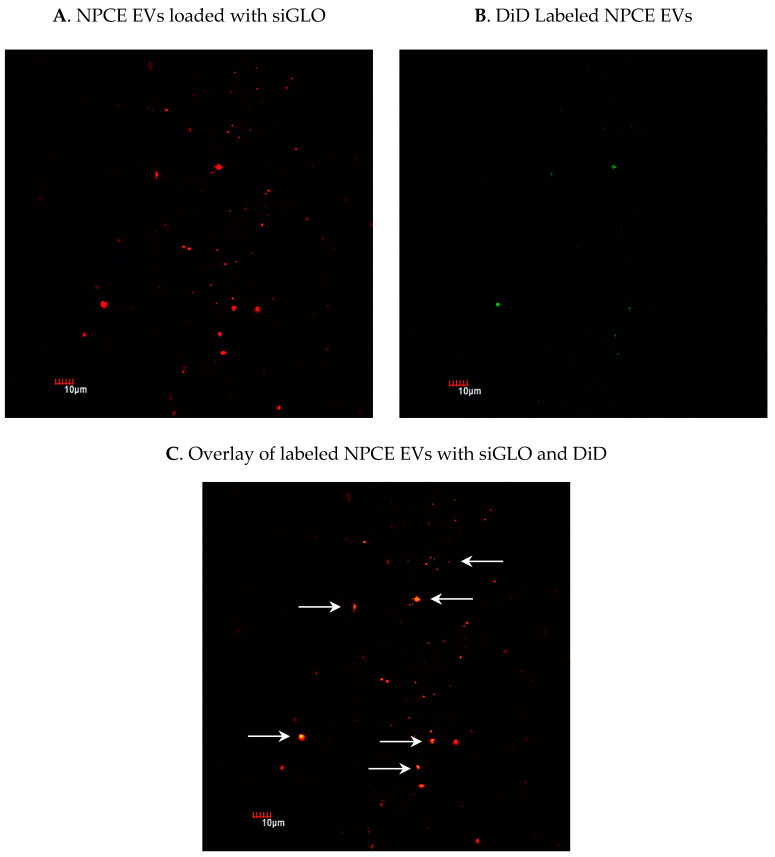
Using confocal microscopy to detect fluorescent siRNA in labeled NPCE-derived EVs and to track the loading efficiency of siGLO to NPCE EVs following electroporation. Purified NPCE EVs were labeled with fluorescent non-coding siRNA, siGLO (green) and with the membrane dye DiD (red). Pictures of NPCE EV’s membrane labeled with DiD (**A**) and NPCE EVs loaded with siGLO (**B**) were integrated (**C**) to determine the percentage of NPCE EVs positive for both DiD and siGLO markers. A total of 10 pictures from 3 different slides were analyzed for each treatment conducted in three independent experiments.

When aiming to use endogenous EVs as nano-sized carriers for the delivery of siRNA, it is important to have an efficient siRNA loading method. To confirm that siRNA was loaded to EVs and exclude siRNAs clusters formation, EVs were pelleted after electroporation. The amount of siRNA fluorescence in the pellet was assayed by confocal microscopy. The percentage of NPCE EVs loaded with siGLO (Figure 3A) from total EVs labeled with DiD (Figure 3B) was evaluated. Results (Figure 3C) indicate a 23.77% encapsulation of siRNA in EVs and no aggregates, using 1 mM EDTA, 50 mM Trehalose, and one unit of RNase H.

### 2.4. TM Cell Viability Following Electroporation

**Figure 4:**
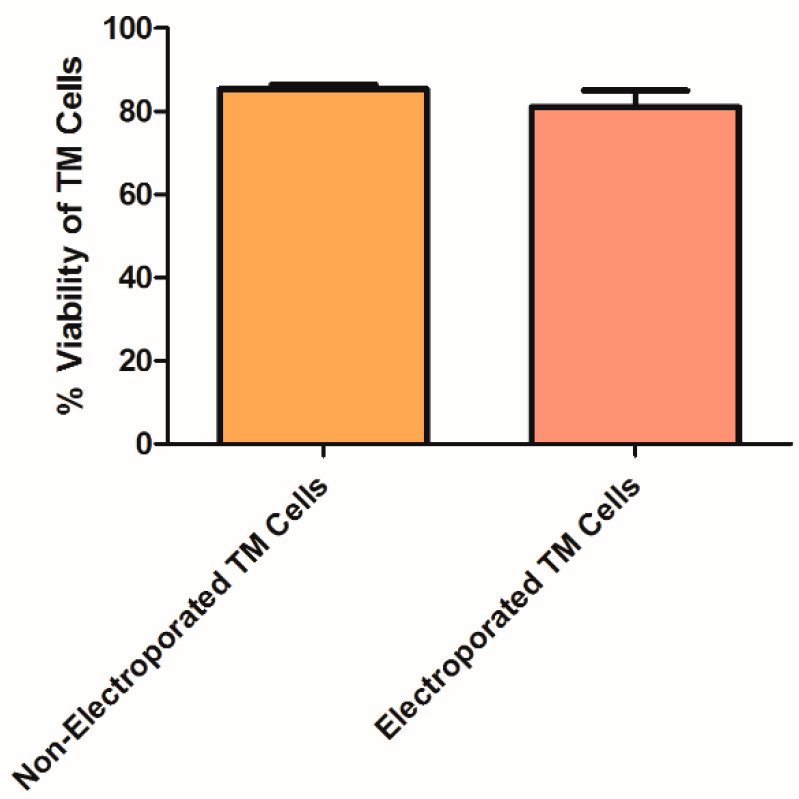
Trypan Blue analysis for the determination of TM cell viability after electroporation. TM cells staining with Trypan Blue dye for the detection of live cells among total cells. Two treatments were examined: Non-electroporated TM cells and electroporated TM cells to estimate the contribution of the electric pulse to cell death. Data represent means ± SD from three independent experiments performed in triplicates. Student’s *t*-Test was used to determine statistical difference.

Since electroporation has been shown by various groups to be the optimal method for loading EVs with siRNA leading to gene silencing and cell toxicity [58,59,62,64], we examined whether the designed siRNA was functional. Positive TM cells loaded with fluorescence siRNA were detected by fluorescence analysis, as previously described (Figure 2). Next, to demonstrate that the electric pulse of 2500 µF and 170v did not lead to extensive cell death, a Trypan Blue assay was conducted. No significant differences in viability were observed between non-electroporated and electroporated TM cells (Figure 4).

### 2.5. siRNA Loading Efficiency to TM Cells Analysis by qRT-PCR

The siRNA reduced SMAD7 transcript levels by 53%. Twenty-four hours post-electroporation compared to untreated TM cells. Comparison of relative mRNA levels of untreated TM cells (1 ± 0.454) to electroporated TM cells (3.146 ± 1.315) indicated that the electric pulse itself contributed to gene activation. Electroporation of non-targeting siRNA to TM cells did not cause a change in SMAD7 mRNA levels compared to untreated TM cells. Data were normalized to the average mRNA level of GAPDH (Table 3).

### 2.6. Delivery of siRNA to TM Cells via NPCE-Derived EVs

**Figure 5:**
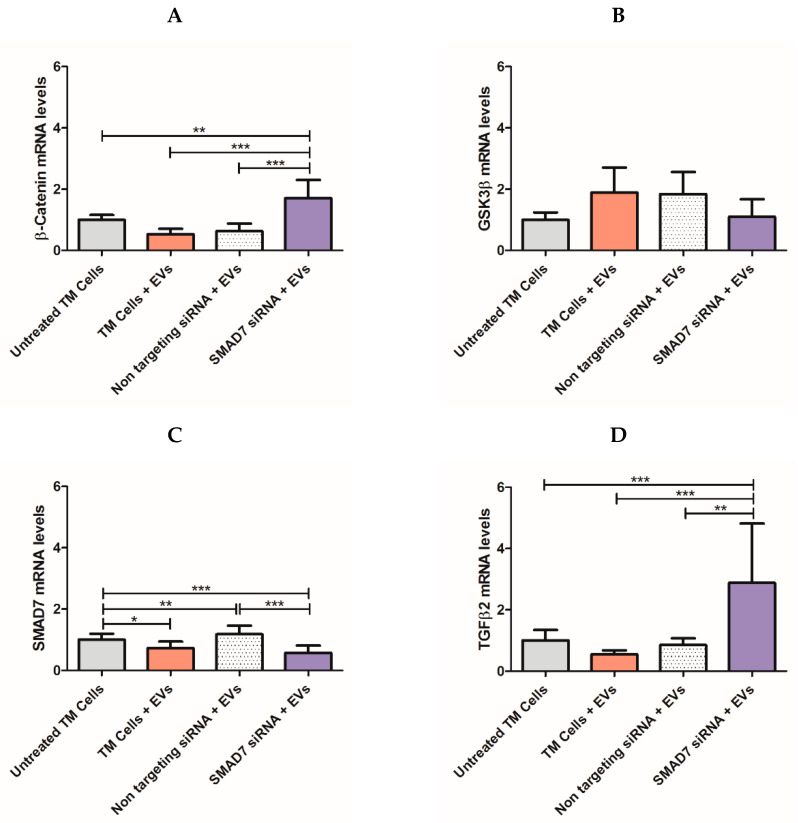
mRNA expression levels of Wnt-TGFβ2 signaling components using qRT-PCR analysis. Real-time qRT-PCR was performed to examine the mRNA expression levels of canonical WNT-TGFβ2 signaling components, including the Wnt transducers β-Catenin (**A**), total GSK3β (**B**), SMAD7 (**C**), and TGFβ2 (**D**). Data were normalized to the average mRNA level of GAPDH. One-way ANOVA analyses were used to study the effect of different TM cells treatments on Wnt-TGFβ2 genes expression. Data represent means ± SD from three independent experiments performed in triplicates. One-way ANOVA was used to determine statistical difference, as indicated by asterisks (* *p* < 0.05, ** *p* < 0.01, *** *p* < 0.001).

Next, we determined whether the siRNA, delivered vie NPCE EVs specifically targets the SMAD7 gene. Relative mRNA levels of TM cells exposed to electroporated NPCE EVs with SMAD7 siRNA were examined using qRT-PCR analysis to measure the down regulation of target genes of the Wnt-TGFβ2 pathway (Figure 5). A mixture of 10 μg NPCE EVs and 10 µg siRNA was used along with 2 × 10^6^ TM cells. A significant decrease (*** *p* < 0.001) in the mRNA levels of TM cells incubated with NPCE EVs loaded with siRNA against SMAD7 (Figure 5C) was detected as compared to untreated TM cells. Moreover, a significant increase in mRNA levels of β-Catenin (Figure 5A) and TGFβ2 (Figure 5D) was revealed compared to those of untreated TM cells, TM cells treated with NPCE EVs, and TM cells incubated with non-targeting siRNA. Consistent with previous findings in our lab [37], a significant decrease (* *p* < 0.05) in mRNA levels of treated TM cells with NPCE EVs compared to untreated ones was seen for the target gene SMAD7 (Figure 5C). The same trend of increase mRNA values was detected for β-Catenin (Figure 5A) and TGFβ (Figure 5D). A significant decrease (* *p* < 0.05) in mRNA levels of treated TM cells with NPCE EVs compared to untreated ones, was seen for the target gene SMAD7 (Figure 5C). For all genes, no difference was found between TM cells treated with NPCE EVs and TM cells treated with NPCE EVs loaded with non-targeting siRNA in the tested gene expression.

### 2.7. Tracking the Expression of Wnt-TGFβ2 Proteins in TM Cells Using SDS-PAGE Separation

**Figure 6:**
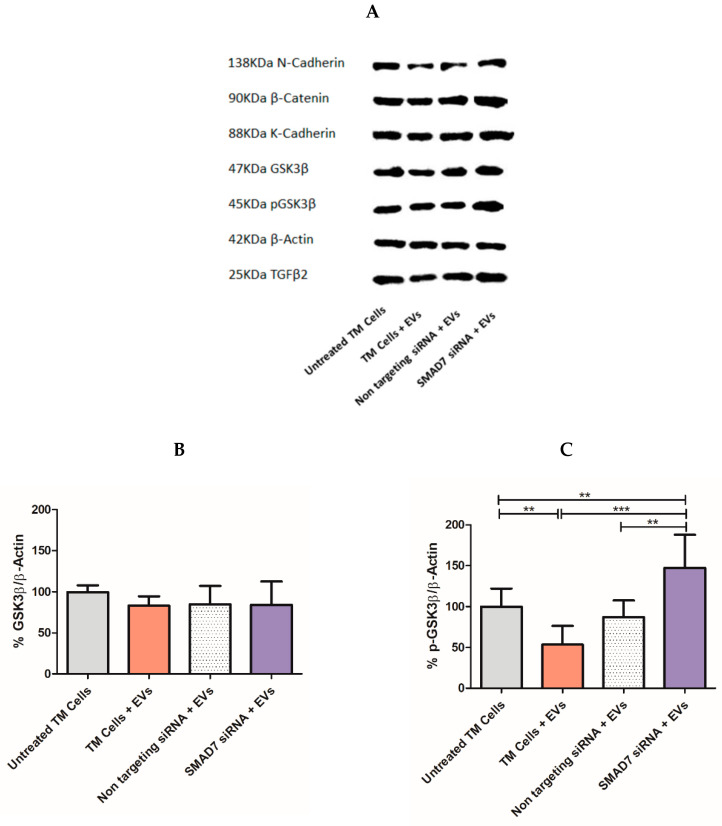

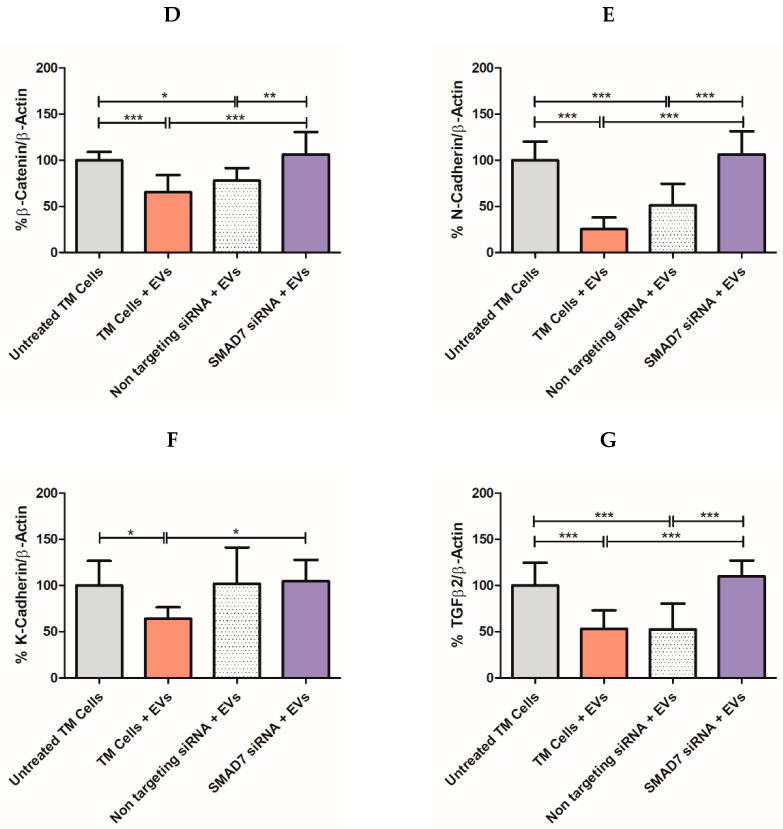
Expression of Wnt-TGFβ2 signaling proteins in TM cells following treatment with NPCE EVs loaded with siRNA. Expression of Wnt-TGFβ2 signaling proteins in TM cells following treatment with NPCE EVs loaded with siRNA. Representative Immunoblots of (**A**) N-Cadherin (138KDa), β-Catenin (90 KDa), K-Cadherin (88KDa), GSK3β (47KDa), p-GSK3β (45 KDa), and TGFβ2 (25KDa) proteins levels normalized to β-Actin (42 KDa) after 24 h incubation of electroporated NPCE EVs with TM cells. The bar graphs of N-Cadherin (**E**), β-Catenin (**B**), K-Cadherin (**F**), GSK3β (**D**), p-GSK3β (**C**), and TGFβ2 (**G**) represent the means ± SD from three independent experiments performed in triplicates. One-way ANOVA was used to determine statistical difference, as indicated by asterisks (* *p* < 0.05, ** *p* < 0.01, *** *p* < 0.001).

Further research of the downstream effects on protein levels in TM cells following incubation with siRNA loaded NPCE EVs was done by Western blot analysis. Twenty-four hours after electroporation of NPCE EVs with SMAD7 siRNA, TM cells were lysed to extract total cell proteins, and the expression of Wnt-TGFβ2 proteins (N-Cadherin, β-Catenin, K-Cadherin, GSK3β, p-GSK3β, and TGFβ2) was determined (Figure 6A). A significant decrease (** *p* < 0.01) in p-GSK3β (Figure 6C) and β-Catenin (*** *p* < 0.001) (Figure 6D) expression levels in TM cells was detected following treatment of NPCE EVs. Exposure of TM cells to NPCE EVs loaded with SMAD7 siRNA caused a significant increase (*** *p* < 0.001) in N-Cadherin (Figure 6G), β-Catenin (Figure 6D), p-GSK3β (Figure 6C), GSK3β (Figure 6B), and TGFβ2 (Figure 6E) levels compared to TM cells treated with NPCE EVs. The effect on the expression levels of K-Cadherin (Figure 6F) in treated TM with NPCE EVs loaded with SMAD7 siRNA was less significant (* *p* < 0.05). In addition, in TM cells incubated with NPCE EVs loaded with SMAD7 siRNA, a significant increase (*** *p* < 0.001) in pGSK3β levels was found compared to untreated TM cells (Figure 6C). No difference was found between TM cells treated with NPCE EVs and TM cells treated with NPCE EVs loaded with non-targeting siRNA for the expression of tested proteins.

## 3. Discussion

Despite the available treatments for POAG disease, glaucoma is still the primary cause for irreversible blindness worldwide with no preventive treatment [2]. Recently, there is growing evidence showing that EVs have neither toxicity nor immunogenicity [49,57,58,65] and thus may act as natural carriers to deliver exogenous nucleic acids and drugs to their target sites [47,56,66]. In the present study, a specific siRNA was introduced into NPCE-derived EVs using electroporation, and was used to transfer siRNA to human TM cells, causing selective gene silencing of SMAD7 in the Wnt-TGFβ2 signaling pathway. siRNA was chosen as the heterologous genetic material, as this double-stranded molecule is small in size and responsible for post-transcriptional gene silencing [64,67]. Electroporation was used to introduce the genetic material into the EVs. Since this method is a physical process that depends on both the field strength and time constant applied, electroporation conditions were optimized with regards to voltage, capacitance, cuvette type, number of electric pulses, and the concentrations of the EVs and siRNA. A literature review revealed that electroporation of EVs with siRNA is accompanied by extensive siRNA aggregates formation [63], which may lead to overestimation of the amount of siRNA actually loaded into the EVs. In addition, electroporation settings, buffers, and equipment may greatly influence electroporation results [60]. The ratio between the amount of EVs and siRNA, and the effectiveness of the silencing percentage in the target cells exposed to engineered EVs, were additional parameters to be optimized [55]. We found that the use of RNAse H and an additional step of ultracentrifugation were useful in preventing the accumulation of siRNA and aggregate formation post-electroporation (Table 2); the average size of the post-electroporated EVs was measured in the range of ~70 nm, which is in correlation with existing data in the literature. Selection of suitable conditions for electroporation as well as an optimal ratio between the EVs and target cells number, made it possible to maintain the physical integrity of the exosomes [65] (Table 3, Figure 1). We achieved a maximal loading efficiency of fluorescent siRNA to NPCE EVs of 23.77%, a rate that is in the top third of loading percentages reported in the literature (Figure 3). The exogenous siRNA could only be detected in the electroporated EVs sample, with a fluorescent value of 20 times greater than the net negative surface charges carried by EVs, siRNA EVs, and siRNA on its own [68,69], ruling out a fusion of siRNA to the surface membrane of EVs [70]. A recently published work demonstrated that surface composition and functionality of EVs to enhance the loading of siRNA by electroporation, can be optimized by the extrusion of lipids compared to the native state of the particles [71]. Collectively, these results provide evidence that the siRNA was effectively introduced into EVs by electroporation.

Next, the functionality of the engineered NPCE EVs incubated with TM cells was tested (Figure 5). A significant (*** *p* < 0.001) silencing of the SMAD7 gene in TM cells (Figure 5C), revealed the specificity and efficiency of the siRNA delivered by the NPCE EVs. Since SMAD7 is an anti-fibrotic regulator of the TGFβ2 receptor, it was expected that knockdown of SMAD7 will lead to an increase in β-Catenin (Figure 5A) and TGFβ2 (Figure 5D). No changes in the mRNA level of GSK3β (Figure 5B) were observed. It can be explained by the fact that in the Wnt signaling cascade, GSK3β is downstream-regulated compared to β-Catenin [7]. The trend of elevation in β-Catenin’s mRNA levels in treated TM cells with NPCE EVs is inversed compared to all other genes due to the role of GSK3β as down-regulator of β-Catenin. The optimal time for siRNA activity detection is 24 h to 96 h [72]. Previous data in our lab suggested that the maximal effect of NPCE EVs on TM cells has two peaks at 2 h [37] and 8 h [9] post-incubation, to detect mRNA or protein levels, respectively. An imaging flow cytometer study used to track the kinetics of NPCE EVs internalization ratio by TM cells resulted in a maximal uptake ratio of EVs to TM cells after 24 h [9]. Since our previous data demonstrated that EVs are mediators of the NPCE-TM cells communication at early time points, in the present study we examined the effects of loaded EVs with siRNA on TM cells at an earlier time point of 24 h rather than 48 h, 72 h, or 96 h. Tracking the expression of Wnt-TGFβ2 proteins in TM cells exposed to NPCE EVs, led to a significant attenuation of the expression levels of all examined proteins (Figure 6) as was previously shown [9,40,41]. Targeting SMAD7 in TM cells with electroporated EVs, eliminated the decrease of both β-Catenin (Figure 6D) and pGSK3 (Figure 6C) proteins levels observed upon addition NPCE EVs to TM cells. Since β-Catenin activates Cadherins and is subjected to TGFβ regulation, the same pro-fibrotic effect after silencing the SMAD7 gene was observed for K-Cadherin (Figure 6F), N-Cadherin (Figure 6E), and TGFβ2 (Figure 6G). For all genes (Figure 5) and proteins (Figure 6), no difference was found between the mRNA’s levels of treated TM cells with NPCE EVs, and those of treated TM cells with NPCE EVs loaded with non-targeting siRNA. Suggesting that the non-targeting siRNA served well as the negative control, with no additional effects on the gene expression that could have been attributed to the electric pulse. Furthermore, each system should be separately calibrated and examined, since the effect of the EVs depends on the uptake mechanism of the target cell, and in the presence of the membrane. Despite electroporation’s prospective usefulness in EVs loading, many aspects regarding the availability of siRNA single-particle electroporation remain to be elucidated. For instance, off-target effects of siRNA introduced by this method and unspecific gene activation induced by the electric pulse (Figure 5) may influence cell function [73]. Therefore, the parameters of single-particle electroporation should be optimized for each cell type [74]. In the present study, we demonstrated a specific delivery of siRNA against SMAD7 gene into TM cells through targeted NPCE EVs that modulated genes and proteins expression related to the WNT-TGFβ2 signaling pathway. Bucolo C. et al. and Pignatelli, F. et al. successfully presented recently the role of TGFβ in ocular diseases [75,76]. It can be concluded that EVs have the potential to serve as vectors for clinical application to lower the expression levels of TGFβ2, owing to their strong biocompatibility. Further investigation of proteins such as collagens, MMPs and TIMPs should be examined in normal TM cells compare to glaucomatous TM cells following exposure to electroporated NPCE EVs. Various microRNAs [77] can be loaded to NPCE exosomes to regulate the expression of genes and proteins involved in inflammatory and degenerative processes. The fine balance-taking place in ECM build-up and deposition is tuned by chronic oxidative stress in POAG-related tissues. However, there is a plethora of challenges ahead to use EVs as drug delivery particles. One such challenge is achieving large-scale production of EVs for clinical use [78]. Furthermore, each system should be separately calibrated and examined, since the effect of EVs depends upon the uptake mechanism of the target cells, and in the presence of specific membrane markers [9,39]. Further in-vivo studies regarding the potency and toxicology of EVs need to be conducted to bring this exciting development a step closer to clinical reality. Nevertheless, the concept of using EVs as delivery vehicles is highly promising [63]. EVs are ideal candidates as biological vectors in gene therapy and should be considered a novel strategy for IOP lowering in POAG patients.

## 4. Materials and Methods

### 4.1. Cell Culture

Cells were cultured according to previously published conditions [9]. Briefly, a human trabecular meshwork (TM) cell line was donated by Alcon Laboratories, Fort Worth, TX, USA, and kept in Dulbecco’s modified Eagle’s medium (DMEM) containing 10% (vol/vol) fetal bovine serum (FBS), two mM L-glutamine, 100 μg/mL streptomycin, and 100 units/mL penicillin (all from Biological Industries, Kibbutz Beit Ha-Emek, Israel) in a humidified atmosphere of 95% air and 5% CO_2_ at 37 °C. The human NPCE cell line was kindly supplied by Prof Miguel Coca-Prados, Yale University, New Haven, CT, USA. TM-5 and human NPCE (ODM-2) cell lines [34] were used in the study. The cell lines authentication test was performed at the Genomics Center of Biomedical Core Facility, Technion, Israel, using the Promega GenePrint 24 System and the results are attached (Figures S1 and S2, Tables S1 and S2). All cell lines were used up to 25 passages. One hundred percent cell confluence was used through the studies. NPCE cells were cultured in DMEM depleted of FBS-derived EVs by overnight centrifugation, using Beckman Coulter ultracentrifugation, for 16 h, 4 °C, and 100,000× *g*. Two hundred milliliters of supernatant were collected and transferred to a 200 mL medium: containing 2 mM L-glutamine, 0.1 mg/mL streptomycin, and 100 units/mL of penicillin. EV-depleted serum was used along with all experiments.

### 4.2. Research Model

To examine whether the attenuation of the Wnt-TGFβ2 signaling pathway in TM cells may be established, NPCE EVs were loaded with SMAD7 siRNA, using a Gene Pulser II System (Bio-Rad Laboratories, Hercules, CA, USA) based on Alvarez-Erviti L., and Momen-Heravi, F. publications [57,65]. NPCE EVs’ integrity following electroporation was evaluated by Cryo-TEM analysis to determine the most suitable conditions for electroporation. According to the literature, the efficiency of siRNAs loading into exosomes is mostly between 0–30% [57,59,62,65,79]. As was reported, unsuitable conditions caused the formation of siRNAs aggregates [57,62,70]. To find appropriate conditions for siRNA loading, with maximal encapsulation, minimal EVs lost, and minimal aggregates formed, modifications in EVs’ concentrations and electroporation parameters were conducted using EDTA, Trehalose, and Ribonuclease (RNAse) H, based on previous publications [65]. EVs were electroporated in four different EV concentrations and pulses. Size and concentration for each condition, including control, were determined using the qNano device. Values represent the mean of three measurements for each condition. Next, the encapsulation of fluorescent siRNA (siGLO, 5 nmole, Dharmacon, Lafayette, CO, USA) into EVs by electroporation was evaluated by confocal microscopy and the infinite m200 (TECAN) device. Aliquots containing electroporated or non-electroporated NPCE EVs were incubated with TM cells in 6-well plates. Twenty-four hours post-electroporation, TM cells were harvested. qRT-PCR was used to verify the transfer of selected siRNA to TM cells assessing the expression levels of the specific miRNAs: SMAD7, GSK-3β, β-Catenin, and TGF-β2. Western blot analysis was used to evaluate qualitative effects on Wnt-TGFβ2 proteins; pGSK-3β, GSK-3β, β-Catenin, TGF-β2, K-Cadherin, and N-Cadherin.

### 4.3. EVs Extraction

EVs isolation was achieved by a series of centrifugations at 4 °C [77]. Briefly, from the supernatant of NPCE cells cultured for 48hr in a medium containing EV-depleted FBS, EVs were extracted. Supernatants (200 mL) of the cell culture were collected and subjected to serial centrifugations, namely 300× *g* for 10 min, 2000× *g* for 10 min, and 10,000× *g* for 30 min. NPCE EVs were then pelleted at 100,000× *g* for 70 min and washed with PBS 0.1M, pH = 7.2, and recovered at 100,000× *g* for 70 min. The pelleted EVs were suspended in PBS 0.1M, pH = 7.2, and stored at −80 °C until use.

### 4.4. Tunable Resistive Pulse Sensing (TRPS)

To determine the optimal EVs concentration for electroporation, and to exclude alternations in EVs size or amount following the electric pulse, EVs at two different concentrations (0.25 µg/µL and 0.5 µg/µL), chosen based on literature, were mixed with non-targeting siRNA (1nmole/mL) and electroporated with one or two electric pulses. Samples containing EVs without an electric pulse were used as a negative control. To eliminate the excess siRNA, the EVs were treated with 1 unit of RNAse H. Before TRPS analysis, EVs samples were passed through 0.22 µm filters to eliminate contaminating debris. Then, NPCE EVs’ size and concentration were determined by the qNano (Izon Science, Christchurch, New-Zealand) instrument, using the Tunable Resistive Pulse Sensing (TRPS) technology with an NP150 membrane (85–300 nm) [80]. The apparatus was operated at a voltage of 0.48–0.64 V without pressure. The membrane was stretched to 47 mm. Polystyrene beads at a concentration of 1.2 × 10^13^ beads/mL (110 nm; Izon Science) were used to calibrate size and concentration, following the manufacturer’s instructions. Samples were diluted 1000-fold with PBS 0.1 M buffer, pH 7.2, and measured up to 10 min. The movement of the particle through the membrane is identified as a change in the ionic stream causing current changes. The signal power is proportional to the particle size. According to the number of particles and their velocity at a specific time, the qNano determines EV’s sizes and concentration.

### 4.5. Cryo-TEM

Evaluation of NPCE EVs membrane integrity following electroporation was conducted using Cryo-transmission electron microscopy (TEM) analysis. Untreated EVs, and four conditions of electroporated EVs in one of two concentrations (0.5 µg/µL vs. 0.25 µg/µL), and a different number of pulses (1 vs. 2), were examined. From each condition, EVs in a concentration of 10^7^ particles/2.5 μL PBS 0.1M, pH = 7.2, were directly adsorbed onto glow-discharged carbon grids with holes blotted at 95% humidity and plunged into liquid ethane. Samples were imaged at liquid nitrogen temperature using a Tecnai 12 TWIN TEM transmission Cryo-electron microscope, operating at 200 kV (JEOL, Japan). A minimum of 50 digital images was taken for each group, using a cooled slow-scan CCD camera (GATAN, Abingdon, UK). Normal or damaged-shaped EVs were evaluated as a percentage of the total particle population in each image.

### 4.6. EVs Labeling with DiD

Purified NPCE-derived EVs were labeled with 2 mM of DiD; a lipophilic fluorescent dye (1,10-dioctadecyl-3,3,30,30-tetramethylindodi-carbocyanine,4-chloro-benzenesulfonate salt, Exc/Ems: 663 nm/669 nm, Biotium, Fremont, CA, USA) at concentration of 2.5 µL/mL PBS 0.1M, pH = 7.2, for confocal microscopy. EVs were incubated for 10 min with the DiD solution at room temperature to ensure uniform labeling. After staining, labeled EVs were suspended in PBS (0.1 M, pH 7.2) and pelleted by ultracentrifugation at 100,000× *g* for 70 min at 4 °C to remove the unincorporated dye [79]. The final DiD-labeled EVs pellet was suspended in a proper volume, calculated by the final concentration of EVs representing 10 µg EVs protein (3 × 10^9^ EVs/mL).

### 4.7. Tracking Electroporated EVs Labeled with siGLO

The encapsulation efficacy of siRNA in EVs was evaluated as follow; 10 μg of NPCE EVs were mixed with 10μg of siGLO green transfection indicator (5nmole, Exc/Ems: 488 nm/525 nm, Dharmacon, Lafayette, CO, USA) in a total volume of 400 µL holding 10µg EVs in PBS 0.1 M, 10 µg siRNA, 50 mM Trehalose and 1 mM EDTA). Samples were electroporated in 0.4 cm cuvettes with aluminum electrodes using a Gene Pulser II System (Bio-Rad Laboratories, Hercules, CA, USA). Samples were diluted 10-fold with PBS 0.1 M and centrifuged for 70 min at 100,000× *g* at 4 °C to remove unbound siRNA and RNAse H residues, as will be described later in more detail. Pellets were re-suspended in 200 µL PBS, introduced to 96 wells plate, and siRNA fluorescence was determined using a fluorescence infinite m200 (TECAN) device reader (Infinite 200) to calculate the percentage of encapsulation compared to a sample containing EVs without the electric pulse used as a negative control.

### 4.8. Confocal Microscopy

The percentage of encapsulated siRNAs into NPCE EVs following electroporation was assessed by confocal microscopy. NPCE electroporated EVs loaded with 10 μg siGLO transfection indicator and non-electroporated EVs were labeled with DiD at a final concentration of 5 mg/mL PBS for membrane staining. A total of 4 µL of labeled EVs (3 × 10^9^/mL) were mixed with 5 µL of Fluromount-G mounting media (Southern Biotech, Birmingham, AL, USA), and a drop of 8 µL was transferred to each coverslip. Detection of siGLO and EVs membrane was performed using an FV1000-IX81 confocal microscope (Olympus, Tokyo, Japan) equipped with a 60x objective. Ten pictures from 3 different slides for each treatment were captured and analyzed by MacBiophotonics Image J software (Version 1.53k14).

### 4.9. Electroporation of TM Cells with siRNAs

Prior to electroporation of NPCE EVs with siRNA, a validation of the effectiveness of loading siRNA sequence for targeting SMAD7 in TM cells was done based on work done by Wei Xue et al. [81]. Briefly, TM cells were suspended in Mirus reagent (MC-MIR-50111, Ingenio, Electroporation solution, Madison, WI, USA) to reach a concentration of 1 × 10^6^ cells/300 µL in 0.4 cm cuvette. A total of 2 µM SMAD7 siRNA was directly loaded (without the involvement of EVs) to 0.8 × 10^6^ TM cells by electroporation, using a Gene Pulser II System (Bio-Rad Laboratories, Hercules, CA, USA). Suitable conditions of 170 v, 2500 µF, 85–140msec, and one pulse were used for electroporation. Post-electroporation, 300 µL of TM cells loaded with SMAD7 siRNA were transferred at once to 6-well sterile plates. Each well-contained 950 µL of 20% DMEM, pre-heated medium (37 °C) was added to reach a final concentration of 500 nM siRNA/1.25 mL medium/well. Twenty-four hours later, the growth medium was replaced with fresh medium contains 20% (vol/vol) FBS. To reach maximal effect, 100% confluent TM cells were harvested 48 h post-incubation. mRNA levels were determined by qRT-PCR analysis to estimate whether the attenuation of SMAD7 gene expression was achieved. Untreated TM cells, TM cells + non-targeting siRNA, electroporated TM cells without siRNA, and three different sequences of siRNA target gene were examined.

### 4.10. Electroporation of EVs with siRNA

NPCE-derived EVs were suspended in 0.4 cm cuvettes with aluminum electrodes using a Gene Pulser II System (Bio-Rad Laboratories, Hercules, CA, USA). Human SMAD7 (24482246, SMAD7HSS180976(3), Stealth siRNA, 20 nmole, Invitrogen, Hercules CA, USA) was examined. For every electroporation, the sample volume was fixed at 400 μL, containing 10 μg EVs (equals to 3 × 10^9^ EVs/mL PBS 0.1M, pH = 7.2) and 10 μg siRNA (1:1 *w*/*w*). EVs were suspended in PBS 0.1M, pH = 7.2 with a supplement of 50 mM Trehalose (Sigma-Aldrich, Waltham, MA, USA) and 1 mM EDTA (Sigma-Aldrich). Pre-electroporation tubes containing EVs and siRNA were incubated for 10 min on ice. Suitable conditions of 400 v, 125 µF, 10–15 msec, and one pulse were used for electroporation. After electroporation, all cuvettes were incubated on ice for 30 min. A total of 1 unit of RNAse H was added to each treatment for 5 min at 37 °C to eliminate aggregates formation in the solution. After 20 min, the activity of RNAse H was neutralized by 1 µL of 0.5 M EDTA/400 µL sample. Then, solutions of electroporated or non-electroporated EVs were diluted with PBS 0.1 M, pH = 7.2 to a final volume of 30 mL and were ultra-centrifuged using Beckman Coulter ultracentrifugation rotor SW28, for 70 min, at 4 °C and 100,000× *g* to exclude siRNA or EVs debris and RNase H residues. Pellets were suspended in 100 µLPBS 0.1 M + 1% *w/v* BSA and were added to TM cells by treatments. Untreated TM cells, TM cells + NPCE EVs (non-electroporated) and TM cells + NPCE EVs loaded with non-targeting siRNA (D-001810-01-05, 5 nmole, Dharmacon, Lafayette, CO, USA) were used as control in all experiments.

### 4.11. TM Cell Viability

Trypan Blue protocol [82] was used to determine the percentage of cells that had clear cytoplasm (viable cells) versus cells that had blue cytoplasm (nonviable cells). TM cells were electroporated (conditions of 2500 µF, 170 v, 0.4 cm, 86 msec were set up), and seeded (0.8 × 10^6^ cells/1.3 mL 20% DMEM/well) in 6-well plates. Twenty-four hours post-electroporation, cell viability was assessed by Trypan Blue (Sigma-Aldrich) uptake. All samples were assayed in triplicates, and the viability of each treatment was compared to non-electroporated TM cells.

### 4.12. Incubation of TM Cells with EVs

TM cells were seeded in 6-well sterile plates (0.5 × 10^6^ cells in 1 mL/well) to reach a confluence of 2 × 10^6^ cells on the day of the experiment. Twenty-four hours later, the growth medium was replaced with fresh EV-depleted medium. 100% confluent TM cells were exposed for an additional 24 h to electroporated NPCE EVs with SMAD7 siRNA to examine the effects of engineered EVs on the signal transfer in TM cells. TM cells incubated with electroporated EVs or EVs without the electric pulse and TM alone were used as controls.

### 4.13. Real-Time Quantitative Polymerase Chain Reaction (qRT-PCR)

Total RNA was isolated from TM cells with different EV treatments using an EZ-RNA Kit (Biological Industries, Beit Haemek, Israel) according to the manufacturer’s instructions. RNA quality and quantity were assessed at 260 nm using a NanoDrop2000 Spectrophotometer (Thermo Scientific, Waltham, MA, USA). Equal amounts (1 µg) of RNA were reverse transcribed in triplicates using Quanta bio qScript cDNA Synthesis kit (QuantaBio, USAerly, MA, USA). Changes in mRNA levels of SMAD7, TGFβ2, GSK-3β, and β-Catenin genes were determined by qRT-PCR with an Applied Biosystems Real-Time PCR QuantStudio™ 1 System (Version 1.5.1), using Syber Green Master Mix (Applied Biosystems, Waltham, MA, USA). The human GAPDH gene was used as endogenous control. The mRNA levels were calculated using QuantStudio Design & Analysis Software (Applied Biosystems, Version 1.4). All primers were purchased from IDT (Integrated DNA Technologies, Redwood City, CA, USA), and their sequences are detailed in Table 4. 

### 4.14. Protein Extraction from TM Cells

Following incubation of TM cells with electroporated or non-electroporated NPCE-derived EVs with siRNAs and TM with no treatment as control, TM cells were lysed as established in our earlier works [9]. Briefly, TM cells were lysed with buffer containing 20 mM HEPES (pH 7.4), 150 mM NaCl, 1 mM EGTA, 1 mM EDTA, 10% glycerol, 1 mM MgCl_2_, 1% Triton-X100, and 10 μL of protease phosphatase inhibitors per 1mL buffer. After incubation on ice for 45 min, the lysates were sonicated for 15 min (40% Amplitude without pulses and), centrifuged at 12,000× *g* at 4 °C, and the supernatant was collected. Supernatants were analyzed for protein concentration using the Bradford assay. Absorbance was recorded on a microplate reader at 595 nm (Thermo Max microplate reader; Molecular Devices, San Jose, CA, USA).

### 4.15. Western Blot Analysis

For the evaluation of the expression, levels of Wnt-TGFβ2 signaling pathway proteins, 20 μg of protein extracted from TM cells for each sample were mixed with Laemmli buffer (Bio-Rad, Hercules, CA, USA) containing 0.1% β-mercaptoethanol, boiled for 5 min at 95 °C and separated on a 10% SDS-PAGE gel. Proteins were transferred to a nitrocellulose membrane, blocked in 5% BSA with 0.5% Tween 20, then probed overnight with primary antibodies to detect and analyze the expression levels of pGSK-3β (1:3000, Ser9, 5B3, D85E12, Cell Signaling, Danvers, MA, USA), GSK-3β (1:1000, ab93926, Abcam, Cambridge, UK), β-Catenin (1:3000, D10A8, #8480, Cell Signaling, Danvers, MA, USA), TGF-β2 (1:1000, ab36495, Abcam), K-Cadherin (1:1000, ab227308, Abcam) and N-Cadherin (1:1000, 33–3900, ThermoFisher, Waltham, MA, USA). Membranes were incubated for 1hr at room temperature with anti-mouse secondary antibodies (1:1000, #115-035-003, Jackson) against GSK-3β, N-Cadherin, and TGF-β2 or anti-rabbit secondary antibodies (1:3000, Cell Signaling, Danvers, MA, USA) against pGSK-3β, β-Catenin, and K-Cadherin. β-Actin levels were determined using anti-β-Actin (1:10,000, Sigma-Aldrich). Immune complexes were detected with chemiluminescence reagent (Thermo Fisher, PierceTM ECL Substrate), followed by exposure to Kodak X-ray film (Rochester, NY, USA). Semi-quantitative analysis was carried out for all Western blot experiments using a computerized image analysis system, MacBiophotonics ImageJ software (Version 1.53k14).

### 4.16. Statistics

Data are presented as mean ± standard deviation. Statistical evaluation of one-way ANOVA was performed with GraphPad Prism version 5 software (La Jolla, CA, USA). Differences between groups were tested using Tukey’s test. Two-way ANOVA and Bonferroni multiple comparisons were used to determine a statistical difference in the siRNA fluorescence measurements. Student t-test was performed to determine changes in the cell viability comparison. All tests were considered significant at * *p* < 0.05.

## 5. Conclusions

NPCE-derived EVs, electroporated with SMAD7 siRNA were able to modify TM cells Wnt-TGFβ2 signaling pathway in vitro. These results may be beneficial as a potential approach for medical intervention to treat POAG patients. NPCE-derived EVs, electroporated with SMAD7 siRNA were able to modify TM cells Wnt-TGFβ2 signaling pathway in vitro. These results, aimed at intervening in the AH drainage resistance—the main cause of intraocular pressure. In the present study, we demonstrated a specific delivery of siRNA against SMAD7 gene into TM cells through targeted NPCE EVs that modulated genes and proteins expression related to WNT-TGFβ2 signaling pathway pressure increase, offer a new way of future intervention that requires further in-vivo research in the TM-SC pathway. Charging exosomes with specific siRNAs can be used as local biological therapy subject to the development of an effective form of administration, in addition to or as an alternative to existing therapy.

## Figures and Tables

**Table 1:** Summary of the electroporation’s effects on NPCE EVs size and concentration by TRPS technology.

Parameters	Untreated NPCE EVs	Condition 1	Condition 2	Condition 3	Condition 4
Size ± s.d. (Mean, nm)	76.67 ± 11.06	77.33 ± 26.51	67.67 ± 14.15	66 ± 7.81	74 ± 1.41
Concentration ± s.d. (Particles/)mL	1.38 × 10^12^ ± 1.38 × 10^12^	2.25 × 10^12^ ± 2.3 × 10^12^	7.94 × 10^11^ ± 2.31 × 10^11^	1.63 × 10^11^ ± 4.67 × 10^10^	1.49 × 10^11^ ± 8.45 × 10^10^
Electroporation Conditions					
Voltage (v)	Non-Electroporated	400	400	400	400
Capacitance (µF)		125	125	125	125
EVs loading (µg/)µL		0.5	0.5	0.25	0.25
Pulse		one pulse	two pulses	one pulse	two pulses

EVs concentration and number of pulses affect electroporation efficiency. The size and concentration of NPCE-derived EVs at four electroporation conditions were analyzed compared to non-electroporated NPCE EVs. For each condition, EVs parameters represent the average of three independent measurements.

**Table 2:** Cryo-TEM analysis of NPCE-derived EVs before and after electroporation.

	Untreated NPCE EVs	Condition 1	Condition 2	Condition 3	Condition 4
Mean of Normal Shaped EVs (%)	91.31	76.02	64.16	65.86	72.86
Mean of Damaged EVs (%)	8.69	23.98	35.84	34.14	27.14

Cryo-TEM analysis for the evaluation of normal or damaged-shaped EVs as a percentage of the total particle population in each image. A total of 91.31% of normal shaped EVs were observed for untreated NPCE EVs, 76.02%, 64.16%, 65.86%, and 72.86% of electroporated NPCE EVs in various conditions were determined. A minimum of 50 images for each group were taken.

**Table 3:** qRT-PCR analysis of electroporated TM cells for the assessment of SMAD7 gene silencing.

Treatments	Mean Ct Value (SMAD7)	Relative mRNA Levels (GAPDH)	Relative mRNA Levels
Untreated TM Cells	24.041	23.223	1 ± 0.454
Electroporated TM Cells	25.128	25.976	3.146 ± 1.315
Non-targeting siRNA + TM Cells	24.038	23.941	1.562 ± 0.102
SMAD7 siRNA + TM Cells	26.470	24.575	0.47 ± 0.015

The expression of SMAD7 in TM cells was measured after electroporation of siRNA. Untreated TM cells, electroporated TM cells, and non-targeting siRNA loaded to TM cells were used as negative controls. Mean Ct (cycle threshold) values of TM cells loaded with siRNA against SMAD7 revealed a reduction of 2.5 cycles in SMAD7 expression compared to untreated TM cells.

**Table 4:** Primers for SYBR green-based qRT-PCR.

Human Target Gene (25 nmol)	Primers	
	Forward	Reverse
SMAD7	5′-CCA ACT GCA GAC TGT CCA GA-3′	5′-TTC TCC TCC CAG TAT GCC AC-3′
TGFβ2	5′-AAG AAG CGT GCT TTG GAT GCG G-3′	5′-ATG CTC CAG CAC AGA AGT TGG C-3′
GSK-3β	5′-CCG ACT AAC ACC ACT GGA AGC T -3′	5′-AGG ATG GTA GCC AGA GGT GGA T-3′
β-Catenin	5′-CAC AAG CAG AGT GCT GAA GGT G-3′	5′-GAT TCC TGA GAG TCC AAA GAC AG-3′
GAPDH	5′-GCA CCG TCA AGG CTG AGA AC-3′	5′-GGA TCT CGC TCC TGG AAG ATG-3′

## Data Availability

Data are contained within the article.

## Supplementary Materials

The following are available online at https://www.mdpi.com/article/10.3390/ph14090858/s1, Figure S1: STR profile of Cell line ID: NTM10 & NTM16, Figure S2: STR profile of Cell line ID: NPCE (ODM-2), Table S1: The STR results of Cell line ID: NTM10 & NTM16, Table S2: The STR results of Cell line ID: NPCE (ODM-2).
