# Supplementary material for: Non-Pigmented Ciliary Epithelium-Derived Extracellular Vesicles Loaded with SMAD7 siRNA Attenuate Wnt Signaling in Trabecular Meshwork Cells In Vitro
Source: Pharmaceuticals (Basel). 2021 Aug 27;14(9):858. doi: 10.3390/ph14090858 (PMC8468932; doi:10.3390/ph14090858)

Report for Cell Line Authentication

The Cell line authentication test was performed at the Genomics Center of Biomedical Core Facility, Technion.

The test was performed using the Promega GenePrint 24 System in order to determine short tandem repeat (STR) profile of 23 loci plus Amelogenin for gender determination (X or XY). In addition, the male-specific DYS391 locus is included to identify null Y allele results for Amelogenin. DNA sample from the kit (2800M Control DNA) was included in the analysis and served as positive control for the PCR step. No DNA template was also included as negative control.

The results were analyzed using the 3500xl Genetic Analyzer (Life Technologies) and GeneMapper IDX software. Allelic ladder was included in the run. Importantly, the 3500xl system is calibrated once a year and spectral calibrated once every 3 months by authorized person.

This report includes the STR results obtained for your cell line together with the available STR profile. It is important to note that not all STRs that are included in the kit are published. Usually, we compare the results to the Cellosaurus, a cell-line knowledge resource (Bairoch A., J. Biomol Tech, 2018).

The interpretation of results is based on allele calls for all 24 loci and should aid determine whether your cell line is indeed the cell line in question or has profile characteristics which indicate a change, contamination or misidentification.

The raw data appears in Supplementary Materials.

Results

Table S2: The STR results of Cell line ID: NPCE (ODM-2).

Marker	NPCE (ODM-2)
**AMEL**	X,Y
**D3S1358**	15,17
**D1S1656**	13,14
**D2S441**	11,13.3
**D10S1248**	15,16
**D13S317**	12,14
**Penta E**	8,13
**D16S539**	12,13
**D18S51**	17,18
**D2S1338**	19,22
**CSF1PO**	10,11
**Penta D**	10,13
**TH01**	7,8
**vWA**	16,17
**D21S11**	28,30
**D7S820**	12
**D5S818**	10,12
**TPOX**	8,12
**DYS391**	10
**D8S1179**	12,15
**D12S391**	20,24
**D19S433**	12,13
**FGA**	23,25
**D22S1045**	11

Summary

There is no published profile for line NPCE (ODM-2).

A search through the database gave no results, meaning there is no known line with this profile.

In light of these results, it seems that the tested cell line is unique however, we cannot state whether the tested line is indeed NPCE (ODM-2).

Date: 12.11.19

Doron Fogel, MSc, Application specialist **Signature:** ______________

Liat Linde, PhD, Head **Signature:** ______________

We would appreciate the acknowledgment of the Genomics Center of Biomedical Core Facility, Technion in your published work that utilizes the cell line above.

Figure S2: STR profile of Cell line ID: NPCE (ODM-2).

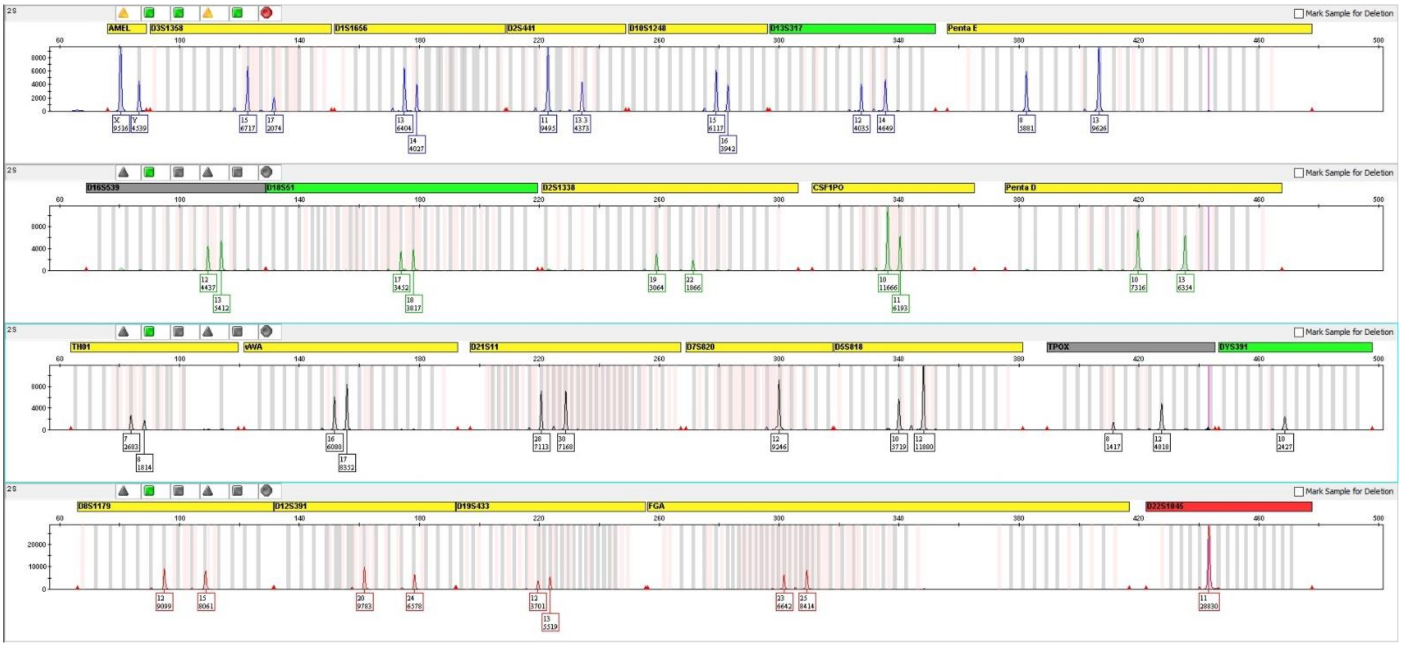

Supplement: Supplementary file 1 [file pharmaceuticals-14-00858-s001.zip › Supplementary Materials-II.pdf]
